# Shifting Conceptualization of Control in Agile Transformations

**DOI:** 10.1007/978-3-030-58858-8_18

**Published:** 2020-08-18

**Authors:** Marius Mikalsen, Viktoria Stray, Nils Brede Moe, Idun Backer

**Affiliations:** 6 IT University of Copenhagen, Copenhagen, Denmark; 7 University of British Columbia, Vancouver, BC Canada; 8 SINTEF, Trondheim, Norway; 9 Norwegian University of Science and Technology, Trondheim, Norway; 10 University of Oslo, Oslo, Norway; 11 Storebrand, Oslo, Norway

**Keywords:** Agile transformation, Agile program, Empirical, Case study, Control, Stewardship theory, OKRs

## Abstract

Agile transformation implies that organizations apply agile methods also outside of software development units. One particular way of doing such transformations is to create cross-functional software development units. This represents new challenges for control for organizations as the unformal agile control mechanisms from the software units meet the more formal, bureaucratic and hierarchical control from other units. The research on how to manage control in agile transformations, however, is scarce. Through a case study of a new, cross-functional unit in a financial institution, we report on their work to implement control in agile transformations. To analyze our results, we draw on new perspectives for control in the digital era, which challenges existing presumptions on control. Our findings indicate how agile transformations require rethinking traditional control mechanisms and experiment with new control perspectives more suitable for the digital era.

## Introduction

The pressure of digitalization with rapidly changing markets and technology developments drive organizations towards adopting agile ways of working, also outside software development units [1]. Such agile transformation implies that agile methods are used not only in software development teams but also by other parts of the organization, such as business units [3]. Agile transformations deal with challenges such as hierarchical management in waterfall mode, difficulties working across organizational boundaries [1], and units not willing or able to change [3]. One particular form of change aiming to overcome some of these challenges is creating semi-independent, cross-functional units (i.e. consisting of personnel from both business- and software-development units) that use agile methods to improve the value of the software developed [2].

Collaboration across different units while working in new ways represent new challenges for control for organizations. The informal agile control mechanisms from the software units meet the more formal and hierarchical control from other units. How to implement control mechanisms that enable management to have control while still allowing the autonomy and rapid changes that are required in agile methods remains an open question in the literature of agile transformations. We, therefore, ask the following research question: *How to manage control in agile transformations?*

To answer this research question, we report from a case study of a financial institution that implements a new semi-independent unit, an agile program, consisting of several cross-functional teams working according to agile principles. The teams consist of both software and business developers. In this paper, we report on their work on devising metrics for measuring the teams’ performance using Objectives and Key Results (OKRs) [11]. To analyze the results, we draw on new perspectives of control for the digital era, based on stewardship theory. Stewardship argues that our conception of software development control needs to be reinvented in an era in which collaborative value creation is increasingly prevalent [4]. Our analysis indicates how agile transformations allow rethinking control and implementing new control perspectives more suitable for the digital era.

The rest of the paper is organized as follows. Section 2 provides the theoretical background on how agile transformation challenges existing control, using stewardship theory as an alternative theoretical perspective. Section 3 introduces the case and explain how we collected and analyzed data. Section 4 shows the findings from one questionnaire and two retrospectives. In Sect. 5, we discuss our findings in light of a stewardship perspective on control. Section 6 concludes and presents future work.

## Theory: A Changing Conceptualization of Control

### Agile Transformation and New Challenges of Control

Agile transformation represents new challenges for control for organizations. Previously, large software development projects were controlled by plans, hierarchies and standardization [5]. As this is no longer suitable, new forms of control are introduced, such as large-scale agile frameworks like SAFe and Spotify, with its own set of challenges [6] that again can challenge autonomy [7, 8]. In the digital era, managers face growing pressure to introduce techniques and practices to improve software productivity and reduce such impediments, however, it is unclear how impediments in software practices are controlled, i.e., identified, measured and managed [9]. One technique many organizations use to guide and measure work is OKRs [11].

### Stewardship Theory – Alternative Conceptions of Control

Recent theoretical work suggests that we need new conceptualizations of control for the digital era. Wiener et al. [4] argue that the digital era, with increasingly advanced and pervasive technologies, changes how we develop software. Changes include: new work practices with blurring of roles, a move from delivering commodities to continuous innovation, a move from hierarchical control structures to leveraging dynamic networks, and a changing workforce with increased specialization. Previous research on control has had an agency perspective, which implied that the purpose of control is to ensure that project actors reduce self-interest and work according to a project, program or organization goal. Now rather, the purpose of control shifts towards value creation, which has support in stewardship theory which considers the agent an intrinsically motivated steward working towards a common overall goal [ibid.].

Wiener et al. [4] outline key control questions and digital-era characteristics based on stewardship theory (see the themes of these two aspects in Table 1 below). First, key control questions are concerned with 1) control modes, whether these are formal or informal, 2) control style, and whether this is authoritative or enabling, and 3) whether there is a value appropriation or value-creation purpose to control. Second, digital era characteristics are concerned with: 1) congruence with the common overall goal. 2) information asymmetry is ok, 3) intrinsically motivated actors, 4) long term orientation rather than short term, and 5) dynamic network structures.

**Table 1.** Aspects of stewardship theory, themes and questions, from Wiener et al. [4].

Aspect of stewardship theory	Theme	Statements
Key control questions	Formal control or informal control	1) We measure what we produce 2) We control each other and that the team delivers
	Authoritative control style or enabling	3) I have extensive dialogue with those who decide the goals
	Value-appropriation or value-creation purpose	4) We measure to handle insecurity regarding budget, time and functionality 5) We measure to handle insecurities regarding collaborations between actors with different competencies 6) We measure to handle insecurities regarding business value
Digital-era characteristics	Congruence with common goal	7) My goals align with the goal of the program
	Information asymmetry	8) It is ok that others (e.g., experts) have information that I do not have
	Intrinsically motivated actors	9) I am intrinsically motivated by working in the program
	Long-term or short-term focus?	10) We focus on short-term goals 11) We consider the development as part of something that continuously changes
	Dynamic network structures?	12) I collaborate with people outside my team

## Case and Method

### Case Background

**Fig. 1.**
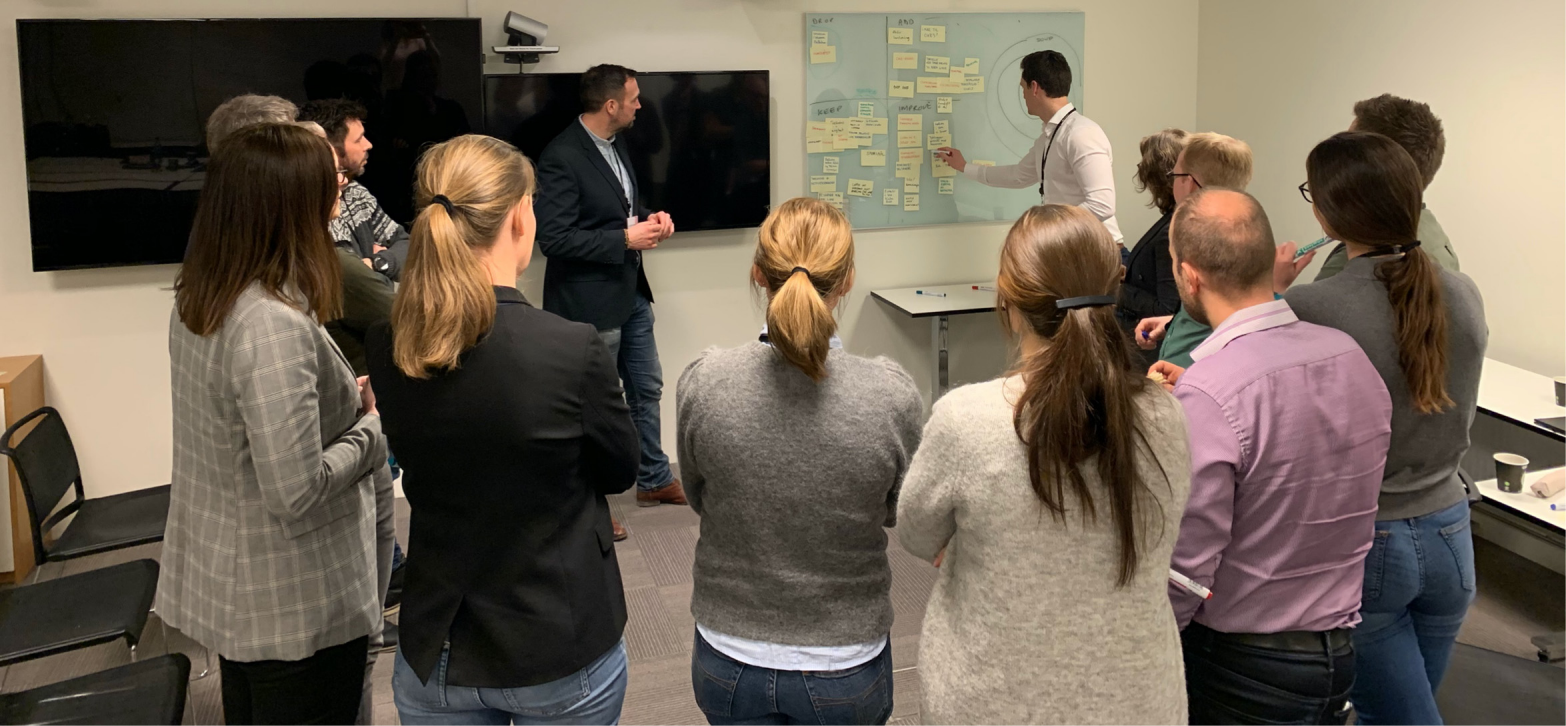
The participants discussing OKR implementation

This study is a part of a longitudinal interpretative case study [10] of agile autonomous teams set in a Norwegian bank (dubbed NorBank for anonymity), with more than 2,000 employees. NorBank initiated in 2017 an agile program (AP) consisting of five cross-functional autonomous teams organized in line with agile principles, with the goal of developing improved software for their business-to-business solutions in the insurance market. The teams consist of resources from both the software and business development side of the organization. The teams deliver software solutions to the business side of the organization, such as sales and settlements. The teams collaborate closely with organizational units responsible for technology development and innovation. Each team is led by product managers, who is part of the steering-forum of the program, together with managers from the business and technology units. The program has been developing software for a while and is now focusing on delivering value on the business side. Their concern is how to measure and control these processes, while still being agile. In response, the program has decided to use OKRs [11] as a method for goal setting and measurement (Fig. 1).

### Data Collection and Analysis

We collected data through two retrospectives with the program in February 2020. The first retrospective was held with four product managers. The second retrospective was with the steering forum of the program. In this retrospective, a total of 12 people participated. The participants included product managers, the leader of the program, managers from the business units, managers from the technology units, and key IT staff such as architects. Each of the sessions lasted about 1.5 h. The authors facilitated the retrospectives and asked questions for clarifications.

Data was collected by taking pictures, documenting the post-its on the whiteboards, taking notes, and collecting data through a questionnaire. Our data analysis was partly deductive as we asked questions regarding stewardship theory in our questionnaire, but also inductive by analyzing our notes and pictures of the whiteboards for themes emerging in the retrospectives. We operationalized the control questions and digital-era characteristics from [4], as shown in Table 1. The statements were given in Norwegian and rated on a 5-point Likert scale from strongly disagree (1) to strongly agree (5). We collected the questionnaire responses digitally during the second retrospective, and the participants answered from their mobile phones. We decided that we wanted to collect the responses this way so that the participants could think individually in silence and also answer anonymously. Furthermore, the tool we used (Mentimeter.com) gave us the ability to show the answers to the questions in real-time, which sparked a discussion among the participants. As such, we were able to get feedback and better understand the responses. The participants scored the statements on a 5-point Likert scale from strongly disagree to strongly agree.

## Findings

### Key Control Questions Regarding Control Configuration, Enactment and Purpose

**Fig. 2.**
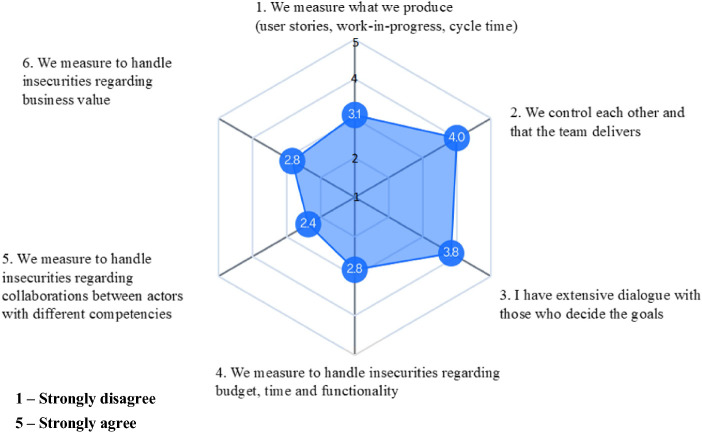
Key control questions regarding control configuration, enactment and purpose

In Fig. 2 below, we see the score on key control questions regarding control configuration, enactment and purpose. Team members control each other (Item 2) and have dialogue with those who decide the goals (Item 3). This is in line with a stewardship perspective on control. Still, we see that traditional forms of measurement such as measuring on time and budget (Item 4) and what is produced (Item 1) are in place, indicating the remains of agency rather than value creation perspectives. Measuring on cooperation between different actors is low (Item 5).

### Findings Regarding Digital Era Characteristics of Control

**Fig. 3.**
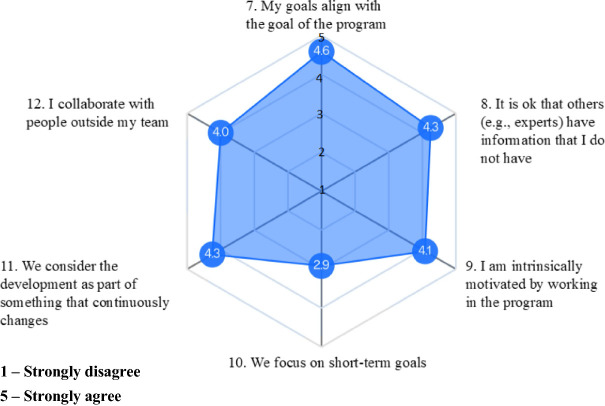
Digital-era characteristics of control

In Fig. 3 below, regarding digital-era characteristics of control, we find scores indicating stewardship assumptions. We see a that practitioners are intrinsically motivated (Item 9). We see that their goals align with program goals (Item 7), that information asymmetry among actors is accepted (Item 8), and there is a lot of cooperation with people outside the team indicating dynamic networks (Item 12). Finally, wee see that there is a is a long-term orientation in the work (Item 11), and a lower score on short-term goals (Item 10).

### Findings from the Retrospectives: Collaboration with Business Units

In the retrospective with the product managers, we found that there were collaboration and interdependencies with units outside the agile program. For example, developers in a separate software development unit made architecture decisions that led to the teams needing to rewrite their APIs. Also, they relied on developers from another unit on implementing workflow automation, and these developers were often busy. Moreover, collaboration with actors outside the organization was challenging. Action items identified included increased use of OKRs and continuous OKR reviews as a way to focus the teams, the need for flexibility from the tech side on delivering on what the teams needed, and also get more competence on workflow optimization.

In the retrospective with the program’s steering forum, the participants discussed that the most important thing to improve was to better demonstrate to the business side the value of what the program delivered. The second most important was OKRs and how this could be used to engage and involve the business side. The third most important was to keep the steering forum meetings lightweight and short. Action items identified included: OKR reviews, quarterly reviews with regard to overall goals, and clarify roles and responsibilities in the steering forum to make sure everyone could contribute more in future meetings.

## Discussion

Agile transformations involve that participants from different units, such as software and business, work together using agile methods [1, 3]. Such collaborations challenge traditional conceptions of control found in agile methods. To answer our research question - *how to manage control in agile transformations* – we have reported findings from a cross-functional unit in the midst of an agile transformation.

We used the stewardship theory [4] to shed light on how a cross-functional unit approach control. Our results show that, regarding control configuration, enactment and purpose (Fig. 2), team members control each other and have dialogue with those who decide the goals. This is in line with a stewardship perspective on control. Still, we see that traditional forms of measurement such as measuring time, and budget and what is produced are in place, indicating the remains of agency rather than value creation perspectives. Measuring cooperation is weaker and can be worth focusing on in order to manage known impediments for flow [9].

Regarding digital-era characteristics of control (Fig. 3), we find strong indication of stewardship assumptions [4]. Practitioners are intrinsically motivated, their goals align with program goals, information asymmetry among actors is accepted, there is a lot of cooperation with people outside the team indicating dynamic networks, and there is a long-term orientation. This indicates that the ways of controlling in the agile program keep with the agile principles of mutual adjustment and autonomy [7].

The retrospectives indicated an appreciation of using OKRs as a way to set goals and communicate goals with the rest of the organization, such as the business units in particular, in order to ensure that what is valuable is agreed upon between units, and that value can be delivered. In terms of stewardship theory [4], such dynamic networks are a key characteristic of the digital era and should also be the focus of new forms of control. It is not clear exactly how to control it. In this case OKRs are used as a way to communicate between the cross-functional teams and the business side. What is clear however, is that determining what is a valuable software deliverable will be an element of negotiation between interdependent units [1].

## Conclusion and Future Work

In this paper, we have used stewardship theory to investigate how cross-functional teams work with OKRs and how new forms of control can emerge in agile transformations. Our results indicate that the members take responsibility for each other and that the team delivers; they also have an extensive dialogue with those who decide the goals. Almost all stated that their goals aligned with the goal of the program, which indicates that the information flow and goal setting works well in this company. The participants also stated that it was ok that others had information that they did not possess. In terms of stewardship theory, this is called information asymmetry. Stewardship assumes that knowledge workers are intrinsically motivated and self-actualizing and that they display a high level of commitment and involvement. The participants in our study reported being intrinsically motivated by working in the program. In sum, our findings indicate the balancing act between new and traditional control in agile transformations.

A key limitation of this study is that it is a single case study. We find indications of stewardship assumptions in the case. However, how and what to measure and control remains a challenge during agile transformations. Future research can investigate how for example, OKRs can be used to support stewardship assumptions rather than older paradigms of control. Also, old paradigms of control are not likely to disappear, so how to incorporate stewardship perspectives with other control regimes is relevant. In sum, agile transformation is about changing practices in organizations, and control seems to us to be a relevant aspect of changing practices.
